# Age-dependent shifts in root resource allocation strategies of *Pinus yunnanensis* seedlings under variable light gradients

**DOI:** 10.3389/fpls.2025.1619386

**Published:** 2025-07-17

**Authors:** Yuanxi Liu, Guihe Duan, Junwen Wu, Rui Shi

**Affiliations:** ^1^ College of Forestry, Southwest Forestry University, Kunming, Yunnan, China; ^2^ Yunnan Provincial Key Laboratory for Conservation and Utilization of In-forest Resource, Southwest Forestry University, Kunming, Yunan, China

**Keywords:** Pinus yunnanensis, light intensity, root nutrient acquisition and storage, biomass allocation, different ages

## Abstract

To elucidate how seedling age affects shade adaptation mechanisms in *Pinus yunnanensis*, we conducted a light intensity experiment with 1- and 3-year-old seedlings under five light levels (100%, 80%, 45%, 30%, and 5% of full sunlight). We evaluated the root non-structural carbohydrates (NSC), carbon:nitrogen:phosphorus (C:N:P) stoichiometry, and biomass allocation using phenotypic plasticity indices and correlation analysis. Phenotypic plasticity analysis revealed distinct age-dependent strategies: 1-year-old seedlings prioritized root morphological features (biomass, surface area) and starch storage, whereas 3-year-old seedlings prioritized metabolic flexibility (soluble sugar/starch ratio, N/P balance). Correlation analyses further demonstrated age-specific resource allocation patterns; in 1-year-old seedlings, biomass was associated with the root C/P ratio and starch reserves, whereas in 3-year-old seedlings, growth was associated with soluble sugars and N metabolism. Investment in photosynthetic organs (needle biomass) was prioritized under shade in 1-year-old seedlings, which is consistent with the source-sink theory; however, the root C content of these seedlings was reduced, and their N uptake was enhanced to maintain chlorophyll synthesis. Conversely, survival was prioritized in 3-year-old seedlings by increasing the amount of structural C in roots and optimizing C:N:P stoichiometry (e.g., lower C/N ratio and higher N/P ratio), which is consistent with the C storage priority hypothesis. These findings highlight a developmental transition from growth-driven C allocation in young seedlings to survival-oriented stoichiometric adjustments in older seedlings, which provides important insights for silvicultural practices in heterogeneous light environments.

## 1 Introduction

Light is an essential driver of plant growth, development, and regeneration (Quero et al., 2006). Its availability and efficient conversion into biomass critically determine tree and stand productivity (Binkley et al., 2013). Forest management and canopy development alter understory light regimes (Forrester et al., 2013), and these dynamics concurrently modify seedling access to light, which affects their spatial distribution and ecophysiological traits (Nelson et al., 2016). The adaptive strategies of seedlings to light heterogeneity are pivotal for forest restoration (Zhou et al., 2017), given that seedlings exhibit heightened sensitivity to environmental factors compared with mature individuals (Soto et al., 2016).

Although moderate irradiance enhances photosynthesis and survival, excessive shading suppresses growth, and intense light triggers photoinhibition, wherein absorbed energy surpasses the photosynthetic capacity, generating reactive oxygen species that damage the photosynthetic machinery (Didaran et al., 2024). Clarifying the responses of seedlings to light gradients is thus vital for elucidating natural regeneration mechanisms and guiding silvicultural practices.

Root systems, which play an important role in water/nutrient acquisition, structural anchorage, and carbon (C) storage (Freschet et al., 2010; Kramer-Walter et al., 2016), profoundly influence seedling performance by affecting biomass allocation, morphology, and stoichiometric plasticity (Guo et al., 2008). Plant root volume, dry weight, total length, surface area, and number of root tips decrease significantly under drought conditions (Wang et al., 2024).

Carbohydrates are the main product of plant photosynthesis and the main energy substance fueling the life activities of plants. They can be divided into two types: structural carbohydrates (SCs), which are the main structural component of plants and play a role in shaping plant morphology, and non-structural carbohydrates (NSC), which are a temporary storage material for the excessive accumulation of photosynthetic products in plants (Kozlowski, 1992) and an energy supply material supporting tree growth (Koch, 1996). Changes in the content of SCs and NSC reflect changes in the metabolic processes of plants, including the balance between C uptake (photosynthesis) and C consumption (growth and respiration) (Poorter and Kitajima, 2007), and the ability of plants to cope with different environmental stresses (e.g., drought, low temperatures) (Würth et al., 2005). Newly synthesized C and assimilates from photosynthesis in plant leaves are transported through vascular bundles and transferred to stalks or roots. When the environment changes, the distribution of NSC in different plant tissues may change, which in turn affects plant growth and responses to environmental changes (Latt et al., 2001). Plants adopt different NSC allocation strategies at different stages of growth and development to fulfill their growth needs. For example, at the early stage of growth and development, plants allocate more photosynthetic products to the underground root system than to the aboveground parts, and the increase in the NSC content in the root system helps absorb more water and nutrients to meet the demand for photosynthesis in the aboveground parts; as the plant grows, the proportion of NSC allocated to the leaves increases, which promotes photosynthesis in plants and provides support (Litton et al., 2004). C, nitrogen (N), and phosphorus (P) are essential nutrients for plant growth and development, and they regulate important physiological and ecological processes such as the photosynthetic rate, transpiration rate, and reproductive growth of plants (Chapin, 1980; Elser et al., 2000); the C content in roots is related to the cost of constructing roots (Guo et al., 2008), and the N content is related to root respiration and longevity (Withington et al., 2006). Study of C, N, and P in roots has implications for plant growth. N and P are important components of plant photosynthetic enzymes, and changes in these elements can affect the photosynthetic rate of plants; consequently, these elements have major effects on plant growth (Liu et al., 2008). More N and P can accumulate in leaves in plants with a higher N and P content in the roots (Chapin et al., 2002; Reich and Oleksyn, 2004).

*Pinus yunnanensis*, a keystone species in Southwest China’s ecological and economic forestry, comprises 19.63% of Yunnan’s forest area (Liu et al., 2023; Zhang et al., 2014). Although the light adaptation of this species has been studied previously (Liu et al., 2024), age-mediated survival strategies under shading, particularly in root plasticity, nutrient acquisition, and NSC dynamics, have not yet been clarified. This study examines 1) differences in growth, biomass allocation, and root nutrient strategies between 1- and 3-year-old seedlings under shading; 2) the relationships between root biomass allocation, nutrient uptake/storage, and whole-plant resource partitioning; and 3) the reliance of older seedlings on reserve mobilization and allometric adjustments for long-term survival. By integrating multi-age experiments with shading gradients, we investigated how *P. yunnanensis* seedlings balance “resource optimization” and “risk avoidance” across developmental stages, which provides profound theoretical insights for artificial afforestation and natural forest management.

## 2 Materials and methods

### 2.1 Study site

The experimental site was located in the arboretum of Southwest Forestry University (Kunming, Yunnan, 25°03′N, 102°46′E). The experimental site is located in a subtropical plateau monsoon climate zone, with an altitude of 1,964 m, average annual temperature of 16.5°C, average annual precipitation of 1,035 mm, and average annual relative humidity of 67%. The temperature inside the shelter was 18.5–37°C, and the relative humidity of the air was 22.3%–48.0%. The soil was a mixture of red loam and humus in a ratio of 3:2. The soil substrate, a 3:2 mixture of red soil and humus, exhibited the following properties: bulk density 1.001 g·cm^-^³, field capacity 22.5%, total C 3.26 g·kg^-^¹, total N 5.98 g·kg^-^¹, total P 0.62 g·kg^-^¹, and pH 7.65.

### 2.2 Plant material and experimental design

*P· yunnanensis* seedlings were grown in Yiliang Garden Forestry, and the 1 and 3-year-old seedlings cultivated in Malonghe Forestry Farm of Shuangbai County (seed number: Yun R-SS-PY-035-2020) were transported to the tree garden of Southwest Forestry University on March 14th, 2024, to cultivate the seedlings prior to transplanting and planting. On March 21, 2024, 1-year-old *P. yunnanensis* seedlings were transplanted into plastic pots with a caliber of 20.5 cm, a base diameter of 14.5 cm, and a height of 18.5 cm, and one *P. yunnanensis* plant was planted in each pot. Three-year-old *P. yunnanensis* seedlings were transplanted into plastic pots with a caliber of 29.5 cm, a base diameter of 23.0 cm, and a height of 21.0 cm. A tray was placed at the bottom of the pot to maintain its soil moisture content at the field water-holding capacity as soon as possible after transplanting to ensure that the seedlings could survive, and the test site was covered with mulch to prevent underground water vapor from affecting the potted plant; this allowed the *P· yunnanensis* seedlings to grow in the suitable environment for 2 months.

A black shade net was used to construct different layers in the shade treatment groups, and the perimeter of the different treatment groups was covered with a shade net to prevent sunlight entering from the side from affecting the experiment. The light intensity in the open field without shade was measured using an auto-ranging illuminance meter (LI-250A, Li-Cor, Lincoln, USA) on a sunny day at approximately noon, and then the shade shed was built to achieve the light intensity required for the different treatments. Our experiment comprised five different light levels (Wang et al., 2006b; Liu et al., 2024): full light (CK, light intensity of 14.69 × 10^4^ to 14.80 × 10^4^ Lx), 80% full light (L1, light intensity of 11.61 × 10^4^ to 12.03 × 10^4^ Lx), 45% full light (L2, light intensity of 6.25 × 10^4^ to 6.74 × 10^4^ Lx), 30% full light level (L3, light intensity of 4.25 × 10^4^ to 4.74 × 10^4^ Lx), and 5% full light level (L4, light intensity of 0.73 × 10^4^ to 0.74 × 10^4^ Lx). The diameter and seedling height of seedlings were measured and recorded on May 21, 2024, for 1-year-old *P. yunnanensis* seedlings (height: 6.69 ± 0.67 cm, diameter: 6.13 ± 0.63 mm) and 3-year-old *P. yunnanensis* seedlings (seedling height: 32.06 ± 2.64 cm, diameter: 15.33 ± 0.98 mm). The experiment was performed using a completely randomized design with three replications of 10 plants each, and there were 150 plants for both the 1-year-old and 3-year-old *P. yunnanensis* seedlings. The shading experiment started on May 21, 2024; the actual soil moisture content was measured using a soil moisture meter and controlled by the weighing method, and rehydration was carried out at 17:00 every day according to the rate of water loss, which stabilized the water within the normal moisture level for each treatment (80 ± 5% of the field-holding capacity, and the actual moisture content was maintained at 36.13%–41.38%). The samples were taken on August 21, 2024, and the experiment lasted for 90 days.

### 2.3 Plant sampling

At the end of the experiment, plant height was measured from the ground to the top of the seedlings using straight-edge (Plant height, accuracy 0.1 cm); diameter was measured using vernier calipers (diameter, accuracy 0.01 mm). Six plants were measured in each treatment, and measurements were taken from a total of 30 one-year-old and 30 three-year-old seedlings. The whole plant harvest method was used to remove the seedlings from the pots; the soil was rinsed with tap water, and the water was removed from the seedlings using filter paper and blotting paper. The *P. yunnanensis* seedlings were divided into three parts, needles, stems, and root system, and the fresh weight of the samples was taken. The root system of *P. yunnanensis* seedlings was placed into a root scanning instrument (Epson) for scanning, and WinRHIZO software was used to obtain data such as the total root length (cm), surface area of the root system (cm^2^), average diameter of the root system (mm), and total volume of the root system (cm^3^); values with two decimal places were retained for all the variables. Subsequently, the plant samples were placed in an oven at 120 °C for half an hour to kill the green living material; they were then subjected to 80 °C and dried to a constant mass, and the dried samples were ground, sieved, and stored. We calculated the needle biomass ratio (needle weight/total plant weight), stem biomass ratio (stem weight/total plant weight), root biomass ratio (root weight/total plant weight), and the root-crown ratio (root weight/(needle weight + stem weight)) based on the weights of the various organs of *P. yunnanensis* seedlings. The water content of each organ was calculated according to the formula for the water content (%) of each plant tissue = (fresh weight - dry weight)/dry weight.

### 2.4 Measurement of plant sample indexes

The NSC in this experiment were the sum of the soluble sugar and starch content; 0.05 g of dried samples were collected and ground; and 10 mL of distilled water was added and centrifuged at 4,000 r-min^-1^ for 10 min after being submerged in boiling water for 10 min. The soluble sugar content in the supernatant was determined using the anthrone method by measuring the absorbance value of the soluble sugar at 625 nm with a UV-visible spectrophotometer; the starch content in the samples was determined by precipitation (Liu et al., 2024). The total C content of needles was determined using the potassium dichromate plus dilution heating method, the total N content was determined by Naïve colorimetry, and the total P content was determined by molybdenum-antimony colorimetry (Bao, 2002).

### 2.5 Statistical analysis

The data (n=6) were assessed for normality and homogeneity (Kolmogorov-Smirnov test) before subsequent statistical analyses. One-way analysis of variance (ANOVA) was used to test the effect of light intensity on the growth and biomass indexes; root indexes; root soluble sugar, starch, and NSC content; and root C:N:P stoichiometric characteristics of *P. yunnanensis* seedlings. Multiple comparisons of means were made using the Duncan test. The variability of each index at two different seedling ages was tested using an independent samples t-test. Analyze the correlations between NSC concentrations in needles and the ratios of C, N, and P elements using R software. All statistical analyses were performed using SPSS 20.0 (IBM SPSS Statistics, U.S.A.), and the level of statistical significance was P = 0.05. Graph Pad Prism 8 was used to make graphs.

Phenotypic plasticity index: PPI =(Xmax-Xmin)/Xmax, where Xmax and Xmin denote the maximum and minimum values of each indicator, respectively.

## 3 Results

### 3.1 Two-factor ANOVA of the growth and root characteristics of *P. yunnanensis* seedlings of different ages under different shading conditions

Under different shading conditions, the growth and root characteristics of 1-year-old and 3-year-old *P. yunnanensis* seedlings differed; differences were also observed among treatments (CK, L1, L2, L3, and L4) (**Table 1**). Significant effects of seedling age were observed on all indicators; however, there was no significant effect (*P*>0.05) of seedling age on the root P content and C/N ratio. There was a significant effect of shade treatment on all indicators; however, seedling age had no significant effect (*P*>0.05) on seedling height, needle-leaf biomass ratio, stem biomass ratio, root biomass ratio, root crown ratio, root water content, and root P content. The interaction of the two (Tree age × Treatment) significantly affected (*P*<0.05) seedling height, needle biomass, stem biomass, total biomass, NSC (and its fractions), C content, N content, P content, C/P, and N/P of *P. yunnanensis* seedlings.

**Table 1:** Two-way ANOVA of different shade treatments on the growth and root characteristic indexes of *P. yunnanensis* seedlings of different ages.

Indicators	Tree age		Treatment		Tree age × Treatment	
	*F*	*P*	*F*	*P*	*F*	*P*
Plant height	396.188	0.000	2.094	0.120	3.639	0.022
Diameter	58.613	0.000	4.945	0.006	1.508	0.238
Needle biomass	165.575	0.000	9.142	0.000	3.183	0.035
Stem biomass	339.888	0.000	11.823	0.000	4.947	0.006
Root biomass	133.508	0.000	5.446	0.004	1.067	0.399
Total biomass	228.964	0.000	10.080	0.000	3.140	0.037
Needle biomass ratio	89.319	0.000	2.301	0.094	2.162	0.111
Stem biomass ratio	72.034	0.000	1.293	0.306	1.009	0.426
Root biomass ratio	12.959	0.002	1.492	0.242	1.326	0.295
Root shoot ratio	13.762	0.001	1.579	0.218	1.366	0.281
Needle water content	81.417	0.000	59.981	0.000	2.245	0.100
Stem water content	121.085	0.000	8.408	0.000	1.099	0.384
Root water content	192.341	0.000	2.841	0.051	1.411	0.266
Root length	59.772	0.000	20.164	0.000	0.585	0.677
Root projected area	54.488	0.000	11.236	0.000	1.499	0.240
Root surface area	54.488	0.000	11.236	0.000	1.499	0.240
Root volume	110.407	0.000	9.405	0.000	0.640	0.640
Root average diameter	25.040	0.000	2.932	0.047	0.772	0.556
Soluble sugar	196.895	0.000	36.950	0.000	10.934	0.000
Starch	45.815	0.000	5.291	0.005	6.902	0.001
NSC	186.201	0.000	29.412	0.000	8.318	0.000
Soluble sugar/starch	28.916	0.000	12.740	0.000	12.808	0.000
C	17.157	0.001	15.768	0.000	5.934	0.003
N	9.921	0.005	15.155	0.000	4.880	0.007
P	0.120	0.733	0.850	0.510	4.455	0.010
C/N	1.356	0.258	17.635	0.000	2.629	0.065
C/P	10.934	0.004	8.130	0.000	14.420	0.000
N/P	8.439	0.009	14.072	0.000	3.531	0.025

F indicates the effect of shade treatment on each indicator; *P*>0.05 means no significant effect, *P*<0.05 means significant difference, and *P <*0.01 means highly significant difference.

### 3.2 Effect of different shade treatments on the growth of *P. yunnanensis* seedlings of different ages

**Figure 1:**
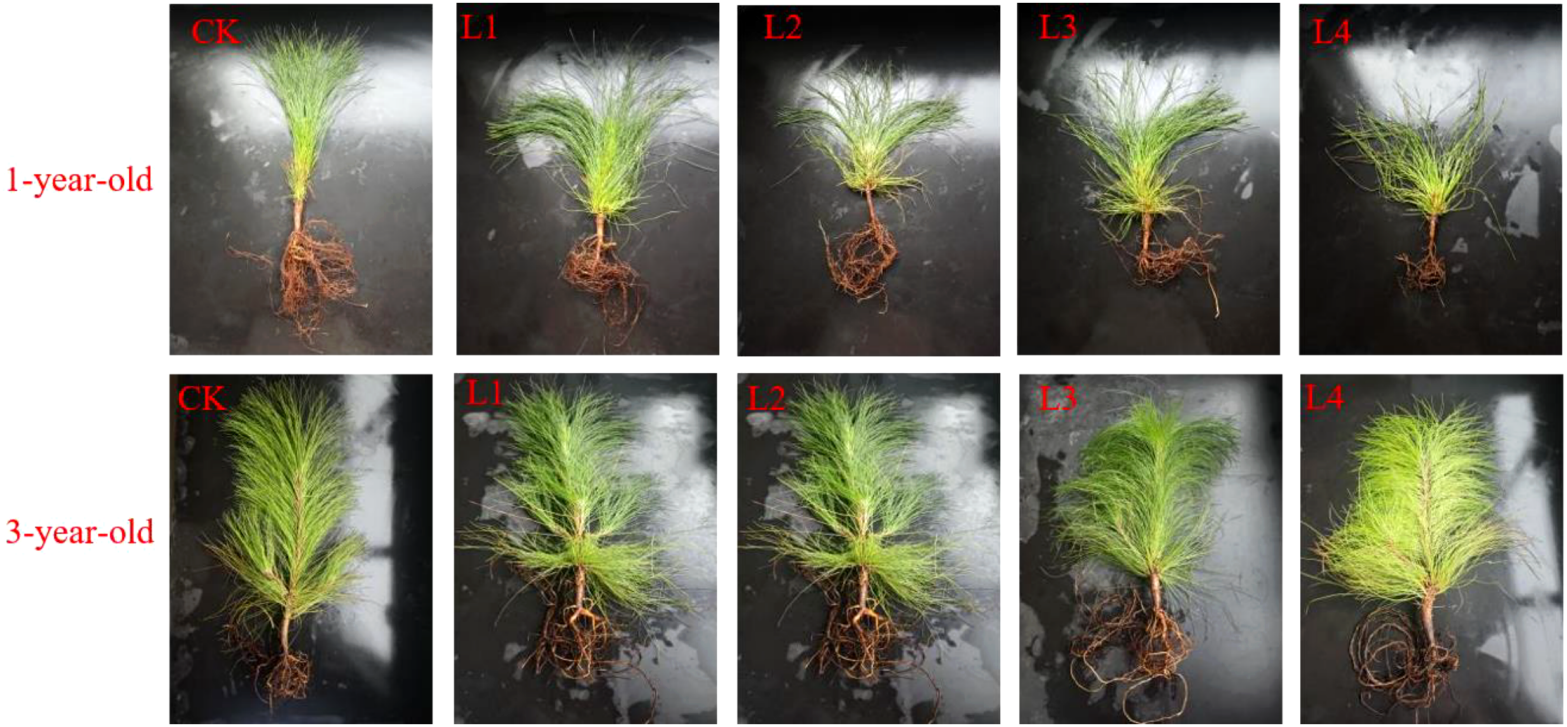
Growth phenotype of *P. yunnanensis* seedlings.

The needles of 1-year-old and 3-year-old seedlings became pendulous after shading, and the needles of shaded plants were not as tough as those of plants in the CK group; the needles of 3-year-old seedlings became yellow and soft compared with those in the control group after shading, and the needles of the 1-year-old seedlings become sparse and yellow (**Figure 1**). The root system of 3-year-old seedlings was significantly more developed in the L3 and L4 treatments than in the L1 and L2 treatments, and the root system of 1-year-old seedlings was also more developed in the L1, L2, and L3 treatments than in the L4 treatment.

**Figure 2:**
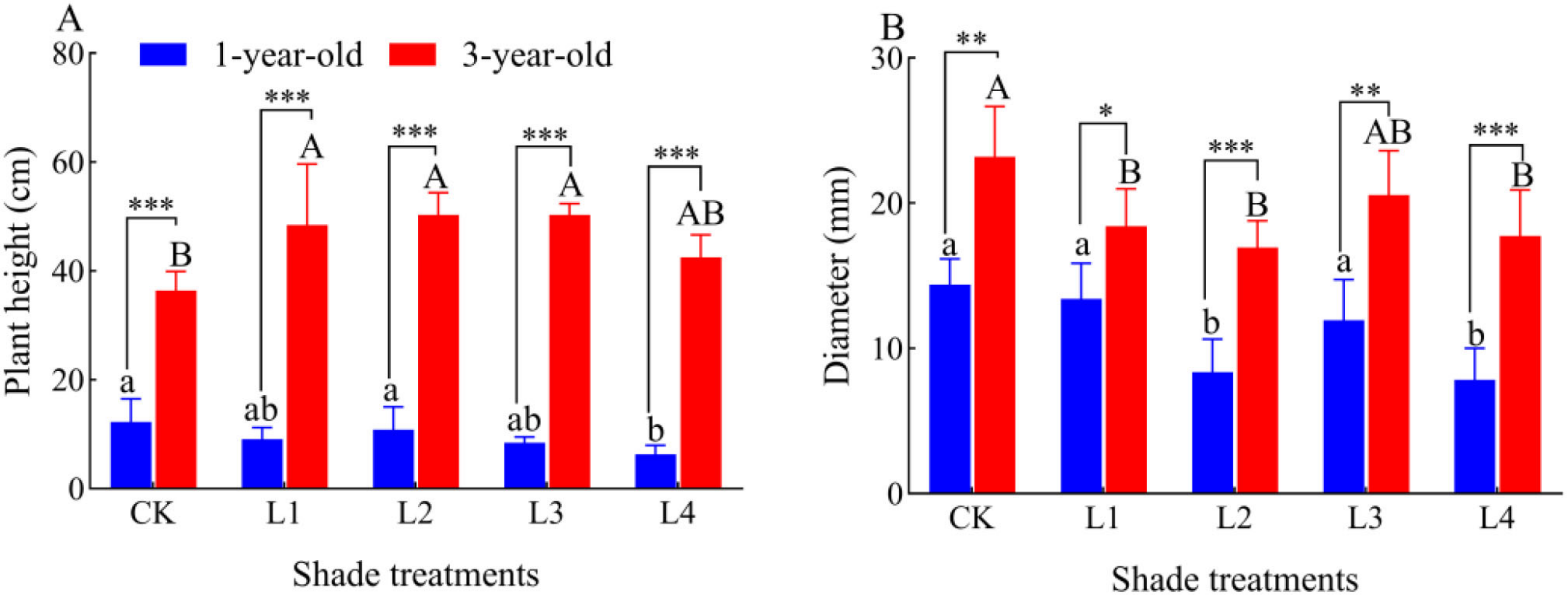
Effect of *P. yunnanensis* seedlings on seedling height and diameter under different shade treatments. CK: full light; L1: 80% full light level; L2: 45% full light level; L3: 30% full light level; L4: 95% full light level. **(A)** Plant height; **(B)** Diameter. The error bars indicate the standard deviation of the mean (n = 6). Different lowercase letters indicate significant differences between treatments for 1-year-old *P. yunnanensis* seedlings, and different uppercase letters indicate significant differences between treatments for 3-year-old *P. yunnanensis* seedlings (*P*<0.05). ***, **, and * indicate significant differences between the 1-year-old and 3-year-old seedlings at *P*<0.001, *P*<0.01, and *P*<0.05, respectively, and the same applies below.

The seedling height of 1-year-old seedlings did not significantly differ between the L1, L2, and L3 treatments and with the CK, and it was 48.36% lower in the L4 treatment than in the CK (*P*<0.05); the seedling height of 3-year-old *P. yunnanensis* seedlings was 33.11%, 38.39%, and 38.40% higher in the L1, L2, and L3 treatments than in the CK, respectively (*P*<0.05) (**Figure 2**). The diameter of 1-year-old *P. yunnanensis* seedlings was significantly more developed in the L2 and L4 treatments than in the CK. The diameter of 1-year-old seedlings was 41.83% and 45.45% lower in the L2 and L4 treatment than in the CK (*P*<0.05), and the diameter of 3-year-old seedlings was 20.65%, 26.97%, and 23.47% lower in the L1, L2, and L4 treatments than in the CK, respectively (*P*<0.05). The height and diameter of 3-year-old *P. yunnanensis* seedlings were significantly higher than that of 1-year-old seedlings (*P*<0.05).

### 3.3 The effect of different shade treatments on the biomass of *P. yunnanensis* seedlings of different ages

**Figure 3:**
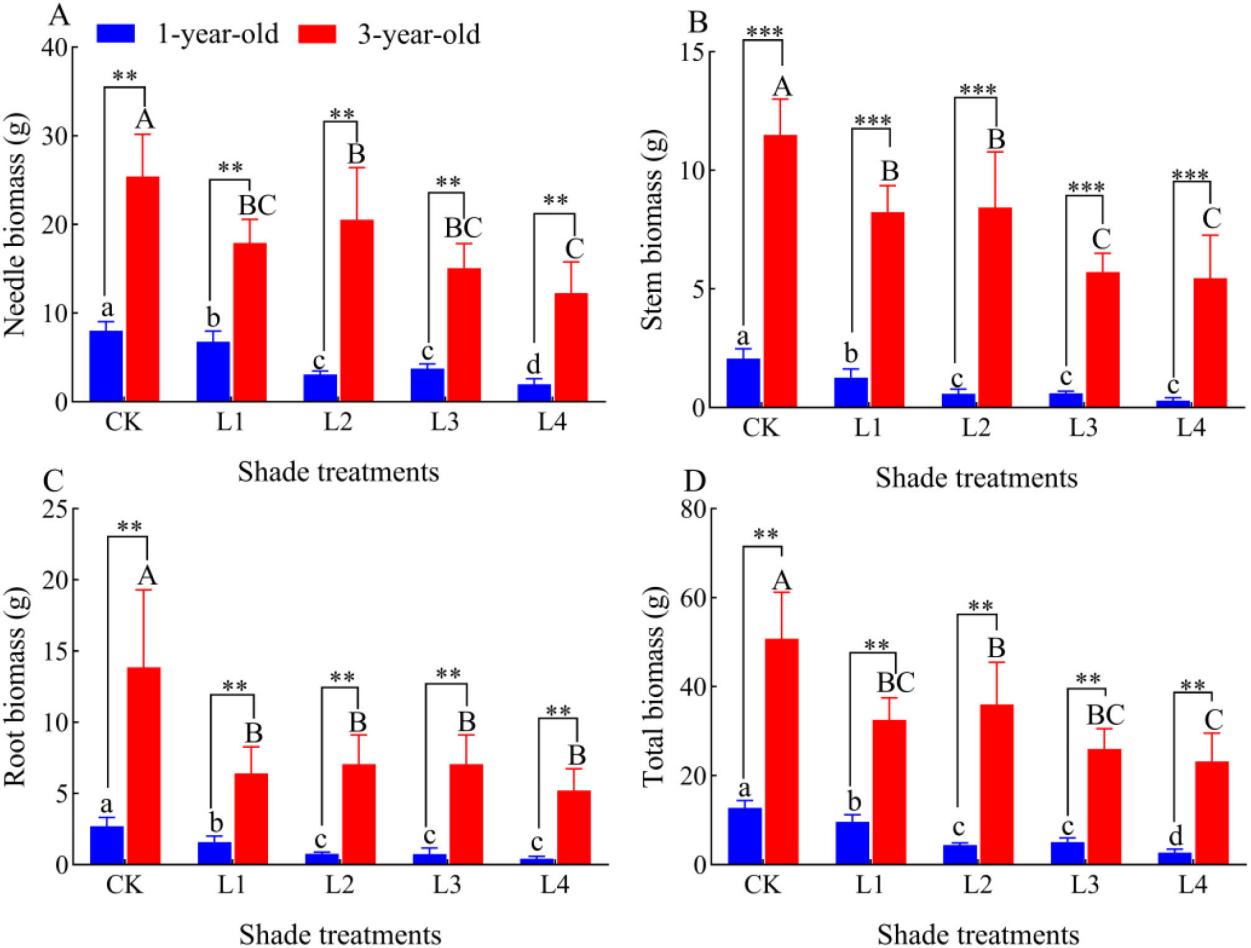
Effect of P. yunnanensis seedlings on biomass under different shade treatments. **(A)** needle biomass; **(B)** stem biomass; **(C)** root biomass; **(D)** total biomass. Different lowercase letters indicate significant differences between treatments for 1-year-old P. yunnanensis seedlings, and different uppercase letters indicate significant differences between treatments for 3-year-old P. yunnanensis seedlings (P<0.05). **and *** indicate significant differences between the 1-year-old and 3-year-old seedlings at P<0.01and P<0.001, respectively.

Needle biomass, stem biomass, root biomass, and total biomass of 1-year-old and 3-year-old *P. yunnanensis* seedlings decreased significantly (*P*<0.05) as the degree of shading increased, and the biomass of 3-year-old seedlings was significantly higher (*P*<0.05) than that of 1-year-old seedlings (**Figure 3**). The needle biomass of 1-year-old *P. yunnanensis* seedlings did not significantly differ in the L2 and L3 treatments (*P*>0.05), and the needle biomass of 3-year-old seedlings did not significantly differ in the L1, L3, and L4 treatments (*P*>0.05). The stem and root biomass of 1-year-old seedlings did not significantly differ in the L2, L3, and L4 treatments (*P*>0.05); the stem biomass of 3-year-old seedlings was significantly higher in the L1 and L2 treatments than in the L3 and L4 treatments (*P*>0.05); the stem biomass of 3-year-old seedlings was significantly higher in the L1 and L2 treatments than in the L3 and L4 treatments (*P*<0.05). The root biomass of 3-year-old seedlings did not significantly differ in the L1, L2, L3, and L4 treatments (*P*>0.05). The total biomass of 1-year-old seedlings was significantly higher in the L2 and L3 treatments than in the L4 treatment; the total biomass of 3-year-old seedlings did not significantly differ in the L1, L3, and L4 treatments (*P*>0.05).

### 3.4 Effects of different shade treatments on biomass allocation in *P. yunnanensis* seedlings of different ages

**Figure 4:**
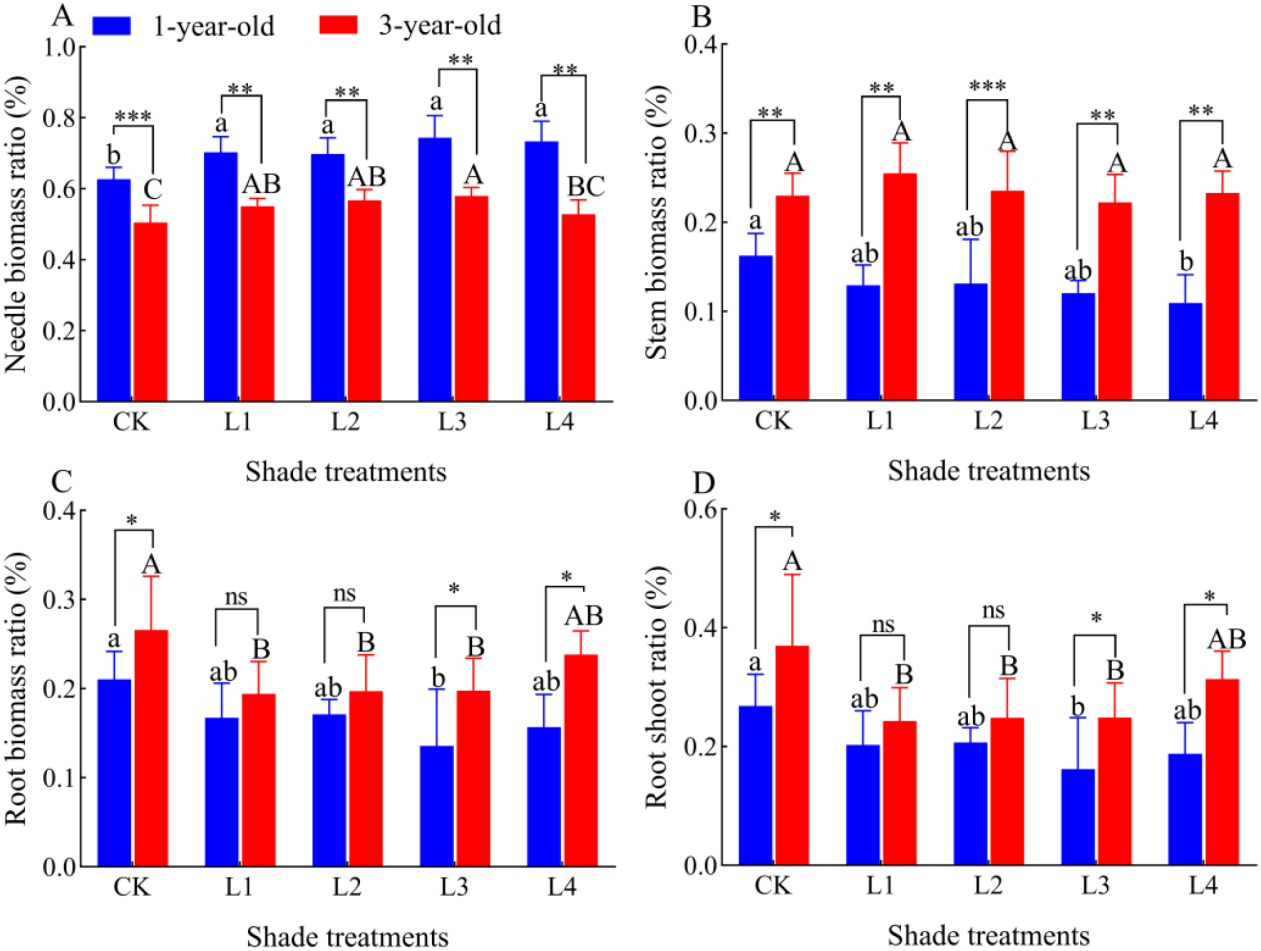
Effect of different shade treatments on the biomass allocation of P. yunnanensis seedlings. **(A)** needle biomass ratio; **(B)** stem biomass ratio; **(C)** root biomass ratio; **(D)** root crown ratio. Different lowercase letters indicate significant differences between treatments for 1-year-old P. yunnanensis seedlings, and different uppercase letters indicate significant differences between treatments for 3-year-old P. yunnanensis seedlings (P<0.05). ***, **, and * indicate significant differences between the 1-year-old and 3-year-old seedlings at P<0.001, P<0.01, and P<0.05;ns indicates no significant difference.

Needle and leaf biomass ratios of 1-year-old and 3-year-old *P. yunnanensis* seedlings increased significantly (*P*<0.05) as the shading degree increased, and the stem biomass ratio, root biomass ratio, and root-crown ratio of 1-year-old seedlings decreased as the degree of shading increased (**Figure 4**). The stem biomass of 3-year-old seedlings did not change significantly as the shading degree increased, and the root biomass ratio and root-crown ratio first decreased as the shading degree increased and then increased in the L4 treatment; however, these ratios were all lower in the L4 treatment than in the CK. The needle biomass ratio of 1-year-old *P. yunnanensis* seedlings was significantly higher than that of 3-year-old seedlings in all treatment groups, and the stem biomass ratio of 3-year-old seedlings was significantly higher than that of 1-year-old seedlings. The root biomass ratio and root-crown ratio were higher in 3-year-old seedlings than in 1-year-old seedlings, and these differences were significant in the CK and L4 treatments.

### 3.5 Effect of different shade treatments on the water content of *P. yunnanensis* seedlings of different ages

**Figure 5:**
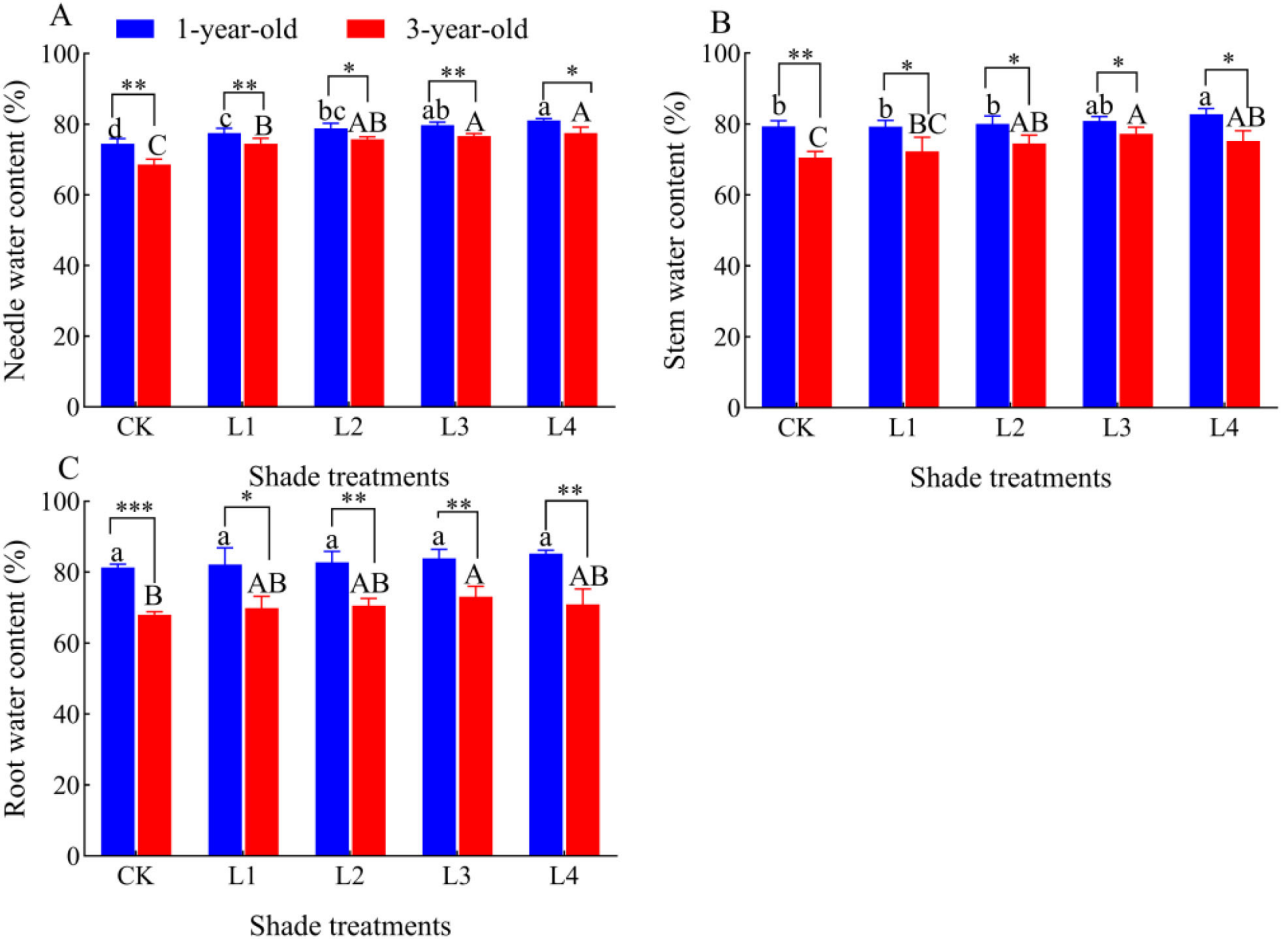
Effect of *P. yunnanensis* seedlings on the water content of each organ under different shade treatments. **(A)** Needle water content; **(B)** Stem water content; **(C)** Root water content. Different lowercase letters indicate significant differences between treatments for 1-year-old P. yunnanensis seedlings, and different uppercase letters indicate significant differences between treatments for 3-year-old P. yunnanensis seedlings (P<0.05). ***, **, and * indicate significant differences between the 1-year-old and 3-year-old seedlings at P<0.001, P<0.01, and P<0.05.

The water content of needles and stems of 1-year-old and 3-year-old *P. yunnanensis* seedlings gradually increased as the shading degree increased; the water content of the roots of 1-year-old seedlings did not significantly differ as the shading degree increased (**Figure 5**). The water content of the roots of 3-year-old seedlings gradually increased as the shading degree increased. The water content of the needles, stems, and roots of 1-year-old *P. yunnanensis* seedlings was significantly higher than that of 3-year-old seedlings (*P*<0.05).

### 3.6 Effects of different shade treatments on the root characteristics of *P. yunnanensis* seedlings of different ages

**Figure 6:**
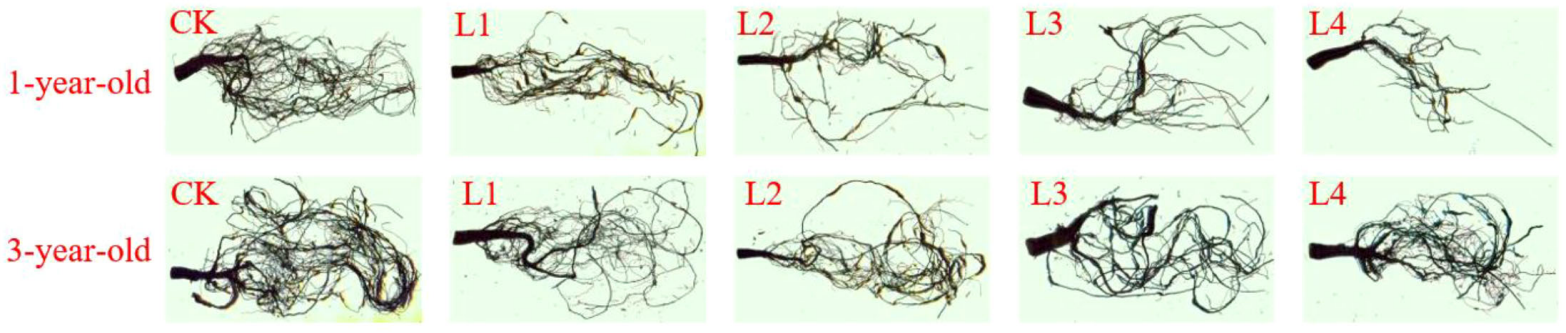
Root phenotypes of *P. yunnanensis* seedlings in different shade treatments.

**Figure 7:**
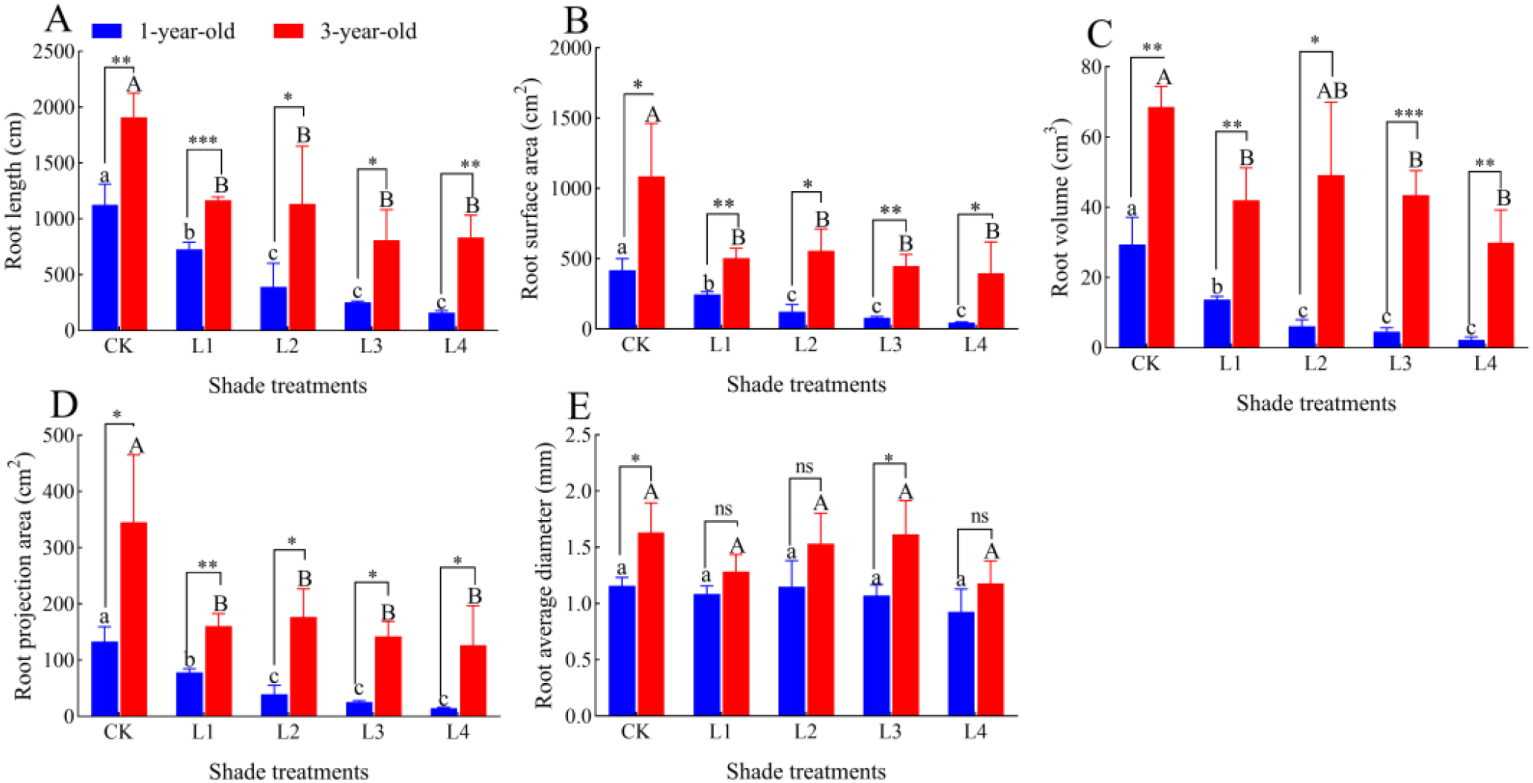
Effect of *P. yunnanensis* seedlings on root characteristics under different shade treatments. **(A)** Root length; **(B)** Root surface area; **(C)** Root volume; **(D)** Root shaded area; **(E)** Root mean diameter. Different lowercase letters indicate significant differences between treatments for 1-year-old P. yunnanensis seedlings, and different uppercase letters indicate significant differences between treatments for 3-year-old P. yunnanensis seedlings (P<0.05). ***, **, and * indicate significant differences between the 1-year-old and 3-year-old seedlings at P<0.001, P<0.01, and P<0.05;ns indicates no significant difference.

The root length, root surface area, root volume, and root projected area of 1-year-old and 3-year-old *P. yunnanensis* seedlings decreased significantly (*P*<0.05) as the degree of shading increased (**Figure 6**; **Figure 7**); the average diameter of the roots did not differ significantly (*P*>0.05) among the treatment groups. All root characteristics of 3-year-old seedlings were higher than those of 1-year-old seedlings. The root length, root surface area, root volume, and root projected area were significantly higher in 3-year-old seedlings than in 1-year-old seedlings (*P*<0.05), and the mean root diameter of 3-year-old seedlings was significantly higher than that of 1-year-old seedlings in the CK and L3 treatment groups; no significant differences were observed among the other treatment groups (*P*>0.05).

### 3.7 Effects of different shade treatments on the root NSC of *P. yunnanensis* seedlings of different ages

**Figure 8:**
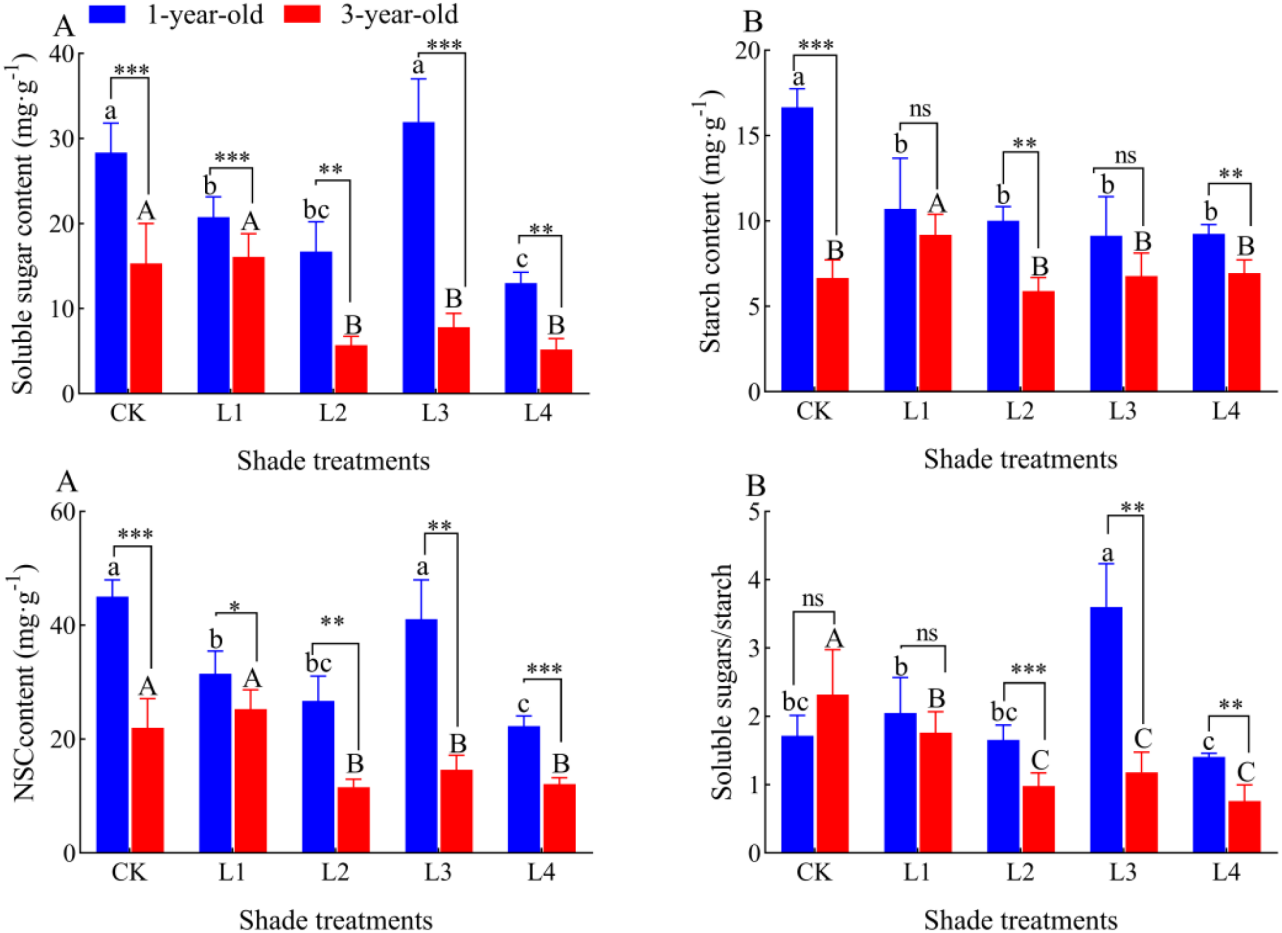
Variation in the root NSC content of *P. yunnanensis* seedlings in different shade treatments. **(A)** soluble sugar; **(B)** starch; **(C)** NSC; **(D)** soluble sugar/starch. Different lowercase letters indicate significant differences between treatments for 1-year-old P. yunnanensis seedlings, and different uppercase letters indicate significant differences between treatments for 3-year-old P. yunnanensis seedlings (P<0.05). ***, **, and * indicate significant differences between the 1-year-old and 3-year-old seedlings at P<0.001, P<0.01, and P<0.05;ns indicates no significant difference.

The soluble sugar content of the roots of 1-year-old *P. yunnanensis* seedlings was 26.84%, 41.03%, and 54.09% lower in the L1, L2, and L4 treatments than in the CK, respectively (*P*<0.05) (**Figure 8**). The maximum value of the soluble sugar content (31.93 mg-g^-1^) was observed in the L3 treatment; the soluble sugar content of 3-year-old seedlings was 62.78%, 48.99%, and 66.19% lower in the L2, L3, and L4 treatments than in the CK, respectively (*P*<0.05). The content of NSC in the roots of 1-year-old and 3-year-old seedlings significantly decreased with the degree of shading. The starch content of 1-year-old and 3-year-old seedlings decreased gradually with the degree of shading. The root NSC content of 1-year-old *P. yunnanensis* seedlings was 30.14%, 40.62%, and 50.54% lower in the L1, L2, and L4 treatments than in the CK, respectively (*P*<0.05), and the root NSC content of 3-year-old seedlings was 47.14%, 34.55%, and 45.65% lower in the L2, L3, and L4 treatments than in the CK, respectively (*P*<0.05). The soluble sugar/starch ratio in 1-year-old seedlings did not significantly differ in the CK, L1, and L2 treatments, and it was highest (3.60) in the L3 treatment. The soluble sugar/starch ratio was 110.05% higher in the L3 treatment than in the CK; it was lowest (1.40) in the L4 treatment, and it was 18.05% lower in the L4 treatment than in the CK in 3-year-old seedlings. The soluble sugar/starch ratio was 24.02%, 57.70%, 49.11%, and 67.14% lower in the L1, L2, L3, and L4 treatments than in the CK, respectively (*P*<0.05). The root soluble sugar content and NSC content were significantly higher in 1-year-old *P. yunnanensis* seedlings than in 3-year-old seedlings. The root starch content was higher in 1-year-old seedlings than in 3-year-old seedlings in all treatment groups, and significant differences were only observed between the CK, L2, and L4 treatments. The root soluble sugar/starch ratio of 1-year-old *P. yunnanensis* seedlings was significantly higher than that of 3-year-old seedlings in the L2, L3, and L4 treatments.

### 3.8 Effects of different shade treatments on the C:N:P stoichiometric characteristics of the roots of *P. yunnanensis* seedlings of different ages

**Figure 9:**
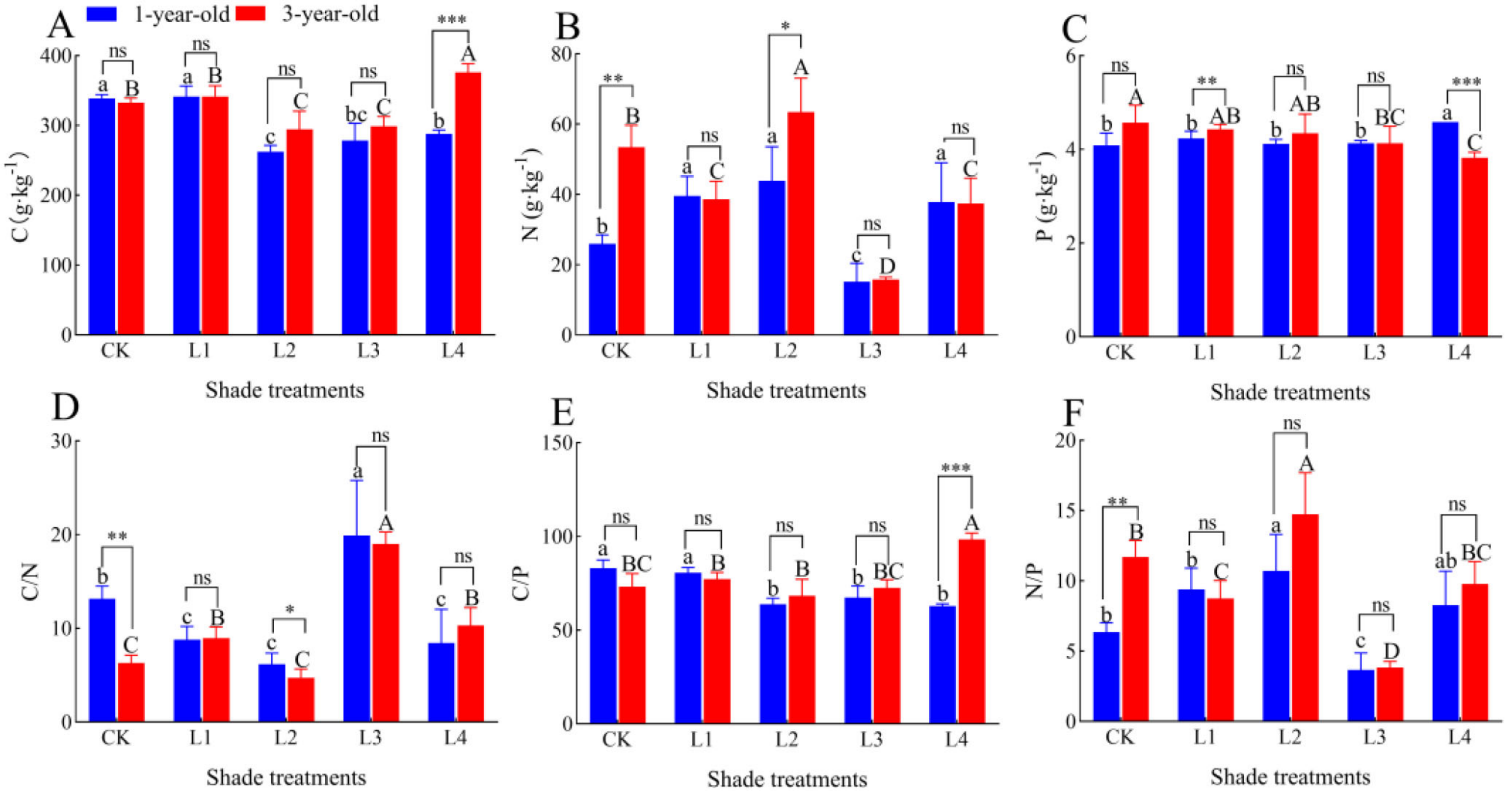
Changes in the root C:N:P stoichiometric characteristics of *P. yunnanensis* seedlings in response to different shade treatments. **(A)** C content; **(B)** N content; **(C)** P content; **(D)** C/N ratio; **(E)** C/P ratio; **(F)** N/P ratio.

The root C content of 1-year-old *P. yunnanensis* seedlings was 22.52%, 17.78%, and 15.02% lower in the L2, L3, and L4 treatments than in the CK, respectively (*P*<0.05); the root C content of 3-year-old seedlings was 11.43% and 12.82% lower in the L2 and L3 treatments than in the CK, respectively (*P*<0.05) (**Figure 9**). The root C content was 24.47% higher in the L4 treatment than in the CK (*P*<0.05). The root N content of 1-year-old *P. yunnanensis* seedlings was 52.63%, 69.32%, and 46.20% higher (*P*<0.05) in the L1, L2, and L4 treatments than in the CK, respectively, and it was 41.61% higher (*P*<0.05) in the L3 treatment than in the CK. The root N content of 3-year-old seedlings was 27.70%, 70.53%, and 70.53% (*P*<0.05) lower in the L1, L3, and L4 treatments than in the CK, respectively. The root N content was 18.73% higher (*P*<0.05) in the L2 treatment than in the CK. The root P content in 1-year-old *P. yunnanensis* seedlings did not significantly differ among the CK, L1, L2, and L3 treatments, and it was 12.09% higher (*P*<0.05) in the L4 treatment than in the CK. The root P content was 9.58% and 9.58% lower in 3-year-old seedlings in the L3 and L4 treatments than in the CK, respectively (*P*<0.05). The root C content of 3-year-old seedlings was significantly higher than that of 1-year-old seedlings in the L4 treatment (*P*<0.05); the root N content of 3-year-old seedlings was significantly higher than that of 1-year-old seedlings in the CK and L2 treatments. The root P content of 3-year-old seedlings was significantly higher than that of 1-year-old seedlings in the L1 treatment, and that of 1-year-old seedlings was higher than that of 3-year-old seedlings in the L4 treatment.

The root C/N ratios of 1-year-old and 3-year-old *P. yunnanensis* seedlings were highest in the L3 treatment, and they were 51.35% and 201.73% higher (*P*<0.05), respectively, in the L3 treatment than in the CK. The root C/N ratios of 1-year-old seedlings were 33.33%, 53.08%, and 36.00% lower (*P*<0.05) in the L1, L2, and L4 treatments than in the CK, respectively. The root C/N ratio of 3-year-old seedlings was 33.33%, 53.08%, and 36.00% lower in the L1, L2, and L4 treatments than in the CK, respectively (*P*<0.05). The root C/N ratio of 3-year-old seedlings was significantly higher than that of 1-year-old seedlings in the L1 and L4 treatments. The root C/N ratio was 42.15% and 63.71% higher in the L1 and L4 treatments than in the CK, respectively (*P*<0.05). The root C/P ratios of 1-year-old *P. yunnanensis* seedlings were 23.20%, 18.81%, and 24.38% lower in the L2, L3, and L4 treatments than in the CK, respectively (*P*<0.05), and that of 3-year-old seedlings was 34.41% higher in the L4 treatment than in the CK (*P*<0.05). The root N/P ratio of 1-year-old *P. yunnanensis* seedlings was 68.58% (*P*<0.05) higher in the L2 treatment and 42.49% lower (*P*<0.05) in the L3 treatment than in the CK; the root N/P ratio of 3-year-old *P. yunnanensis* seedlings was 25.32% and 67.20% lower (*P*<0.05) in the L1 and L3 treatments, respectively, and 25.88% (*P*<0.05) higher in the L2 treatment than in the CK. The root C/N ratio of 1-year-old *P. yunnanensis* seedlings was significantly higher than that of 1-year-old seedlings in the CK and L2 treatments (P<0.05); the root C/P ratio of 1-year-old seedlings was significantly lower than that of 3-year-old seedlings in the L4 treatment (*P*<0.05); the root N/P ratio of 1-year-old seedlings was significantly lower than that of 3-year-old seedlings in the CK treatment (*P*<0.05).

### 3.9 Phenotypic plasticity analysis of growth and root characteristics of *P. yunnanensis* seedlings of different ages under different shade treatments

**Figure 10:**
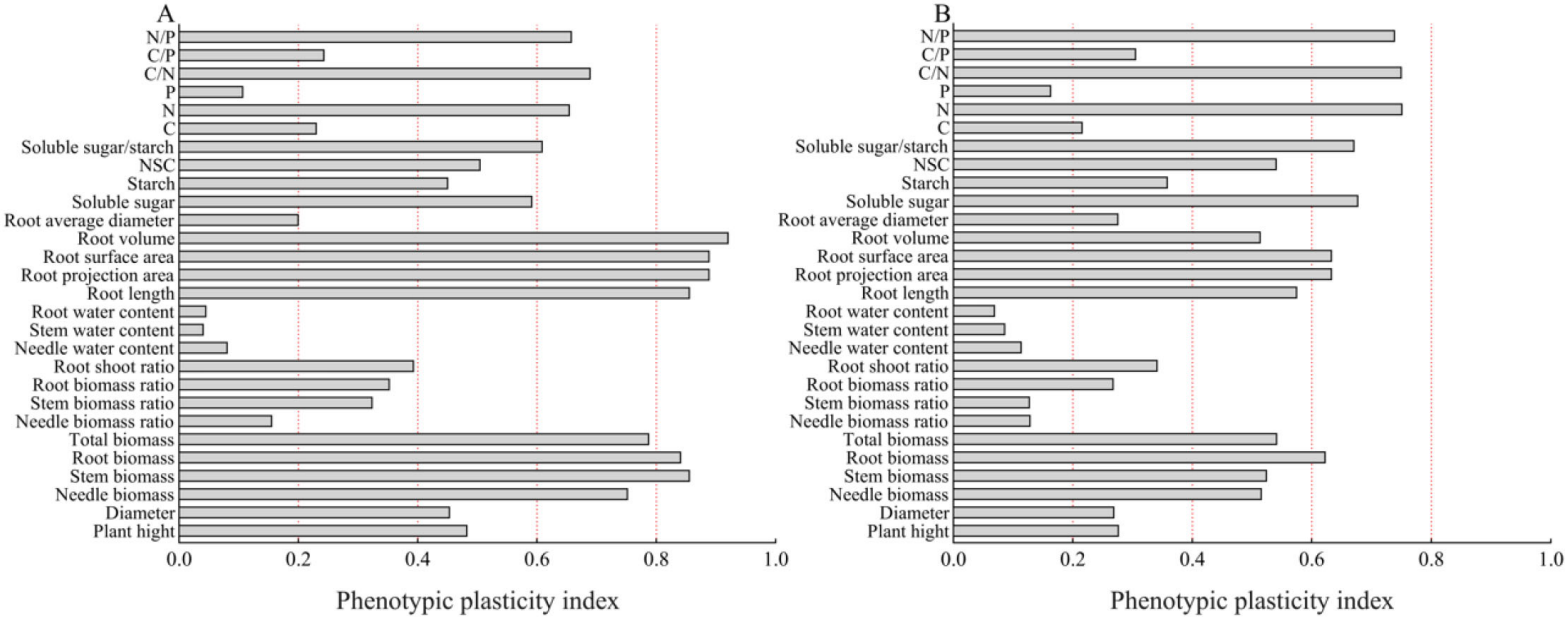
Phenotypic plasticity of growth and root characteristics of *P. yunnanensis* seedlings of different ages under different shade treatments **(A)** 1-year-old; **(B)** 3-year-old.

The phenotypic plasticity indices of various organs in *P. yunnanensis* seedlings of different ages are shown in **Figure 10**; the phenotypic plasticity indices of 1-year-old and 3-year-old seedlings differed among the shade treatments. The phenotypic plasticity indices of 1-year-old *P. yunnanensis* seedlings were larger for root system characteristics, root biomass, and stem biomass (**Figure 10A**), followed by root N/P, C/N, N content, and soluble sugar/starch ratio. The phenotypic plasticity indices of 3-year-old *P. yunnanensis* seedlings were larger for the N/P, C/N, N content, soluble sugar/starch ratio, soluble sugar, root surface area, and root projected area, followed by N/P, C/N, N content, soluble sugar/starch ratio, soluble sugar, root surface area, NSC, root volume, root length, acinar biomass, stem biomass, and total biomass (**Figure 10B**). Overall, this suggests that both 1-year-old and 3-year-old seedlings adapted to the shade environment by altering their root characteristics, root N/P, C/N, N content, and soluble sugar/starch ratio.

### 3.10 Correlation analysis of growth and root characteristics of *P. yunnanensis* seedlings of different seedling ages under shade treatment

**Figure 11:**
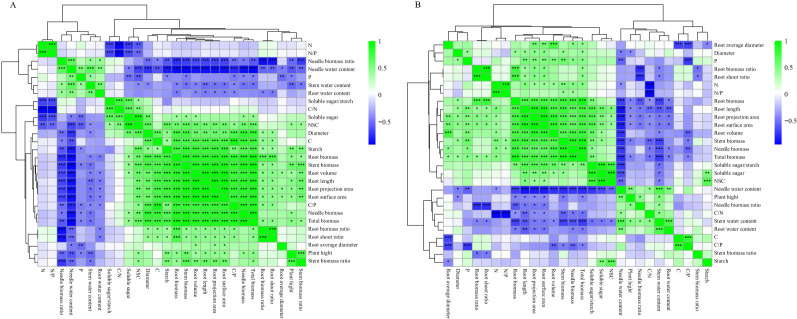
Correlation analysis of growth and root characteristics of P. yunnanensis seedlings under different shade treatments. **(A)** One-year-old seedlings; **(B)** Three-year-old seedlings. *P<0.05, **P<0.01**, *P<0.001.

The correlation between the growth and root characteristics of *P. yunnanensis* seedlings of different ages is shown in **Figure 11**, and the correlation of the indicators under different shade treatments differed for 1-year-old and 3-year-old seedlings. The water content of needles and stems of 1-year-old and 3-year-old seedlings was significantly negatively correlated with the diameter, biomass of each organ, root length, root projected area, root surface area, root volume, and root NSC; the biomass of each organ, the diameter and root length, root projected area, root surface area, root volume, and root NSC content were generally significantly positively correlated. The biomass of each organ of 1-year-old seedlings and the diameter were significantly positively correlated with the root starch, C content, and C/P (**Figure 11A**). These variables were positively correlated with the root starch, C content, and C/P in 1-year-old seedlings (**Figure 11A**), and organ biomass, diameter, root soluble sugar/starch, N content, and N/P were significantly positively correlated in 3-year-old seedlings (**Figure 11B**).

## 4 Discussion

### 4.1 Light and seedling age affect the growth strategy of *P. yunnanensis* seedlings

Most plants adapt to the shaded environment by altering their biomass allocation pattern and morphological indexes to maximize the acquisition of light energy, improve photosynthetic efficiency, and promote their growth (Liu et al., 2018). Changes in the plasticity of seedling height and diameter under different light treatments reflect their ability to utilize resources (Lockhart et al., 2012). In this study, we showed that there was a significant difference in the response of seedling height and diameter to light in the 1-year-old and 3-year-old *P. yunnanensis* seedlings (**Figure 1**). Both the seedling height and diameter of 1-year-old seedlings were significantly decreased under low light (L4, 95% full light); however, the seedling height of 3-year-old seedlings was significantly higher in the shade treatment (38.40% increase in seedling height in the L3 treatment). This pattern of growth of 3-year-old seedlings was similar to that of *Cunninghamia lanceolata* (Lamb.) Hook, where all the growth variables of seedlings increased with the light intensity (Liu et al., 2018); the opposite growth pattern was observed for 1-year-old seedlings. This suggests that as seedlings grow, *P. yunnanensis* may enhance adaptation to shade by altering their photosynthetic capacity or resource allocation strategies. In contrast, the growth in the height and diameter of 1-year-old *P. yunnanensis* seedlings was suppressed, and this “dwarfing” growth may have been achieved by reducing the metabolic cost of stem elongation while increasing the water-holding capacity of leaves, roots, and stems to cope with potential stress (**Figure 5**), which is consistent with the findings of studies on *Pseudotsuga menziesii* (Chen and Klinka, 1997), American chestnut (*Castanea dentata*) (Wang et al., 2006a), *Fagus sylvatica* and *Quercus robur* (Sevillano et al., 2016), and many other tree species. Comparison of the growth indexes of 1-year-old and 3-year-old *P. yunnanensis* seedlings revealed that the adaptation of 1-year-old *P. yunnanensis* seedlings to the shaded environment was alleviated under 30% full light, and 3-year-old *P. yunnanensis* seedlings grew best under 30% full light.

Biomass can reflect plant growth and productivity, and the biomass allocation pattern indicates the adaptation of plants to different light treatments; the allocation of biomass between the aboveground and belowground parts reflects their overall morphological adaptation (Portsmuth and Niinemets, 2007). In this study, the biomass of 1-year-old and 3-year-old *P. yunnanensis* seedlings decreased significantly as the degree of shading increased, and the needle-leaf biomass ratio increased significantly in both 1-year-old and 3-year-old seedlings as the degree of shading increased. This indicates that shade inhibited the accumulation of biomass in young *P. yunnanensis* seedlings, and that resources were preferentially allocated to photosynthetic organs (needles and leaves) under low light (Mediavilla and Escudero, 2010); the needle-to-leaf allocation ratio was significantly higher in 1-year-old seedlings than in 3-year-old seedlings, and the biomass allocation ratios of stems and roots were higher in 3-year-old seedlings than in 1-year-old seedlings. This suggests that 1-year-old *P. yunnanensis* seedlings responded to shade by relying mainly on inputs to needle resources, whereas 3-year-old seedlings responded to the shade environment in an integrated manner through balanced inputs from multiple sources. This is also consistent with our finding that the root biomass ratio, stem biomass ratio, root crown ratio, and root characterization indexes were significantly higher in the 3-year-old seedlings than in the 1-year-old seedlings.

In summary, 1-year-old seedlings compensated for the lack of light energy in low-light environments by prioritizing the allocation of limited resources to photosynthetic organs (needles and leaves). This is consistent with the source-store theory, in which young plants prioritize the maintenance of the activity of “sources” (photosynthetic organs) to ensure basal metabolic requirements when resources are limited (Liu et al., 2020). Moreover, root development is difficult to maintain in young seedlings under low light, and prolonged shading may lead to seedling death due to inadequate root development. In contrast, 3-year-old seedlings can maintain the dynamic balance of underground-aboveground resources and enhance the adaptability to resource heterogeneity by moderately inhibiting aboveground growth while safeguarding root function. By optimizing resource allocation and physiological plasticity, “robust growth” can be achieved under shade. This strategy may provide seedlings with a competitive advantage in secondary forest restoration or sparse plantations, especially in habitats with frequent light fluctuations.

### 4.2 Effects of different light conditions and seedling age on NSC content and C:N:P stoichiometric characteristics in the roots of *P. yunnanensis* seedlings

Changes in the NSC content reflect the relationship between the tree C balance and plant adaptation strategies to environmental changes (Liu et al., 2024), and the allocation of NSC in trees is related to their physiological and ecological processes (Poorter and Kitajima, 2007). The C, N, and P content and their stoichiometric ratios in roots reflect the pattern of C accumulation as well as N and P uptake and storage, which can reflect the relationship between the plant growth rate and nutrient partitioning. Previous studies have shown that when plants are deficient in N, they increase the proportion of carbohydrates allocated to the root system to promote N uptake, whereas an increase in N effectiveness results in the allocation of more carbohydrates to aboveground tissues (Bai et al., 2010). In this study, soluble sugars, starch, and NSC in the roots of 1-year-old and 3-year-old *P. yunnanensis* seedlings decreased significantly as the degree of shading increased; the soluble sugar and starch content of the roots of 1-year-old *P. yunnanensis* seedlings did not significantly differ in the L3 treatment and the control (**Figure 8**), which indicated that the roots of *P. yunnanensis* seedlings responded to the shade environment by increasing the consumption of NSC for transport to other organs (Wei et al., 2022). Under shade, the soluble sugar and starch content of 1-year-old *P. yunnanensis* seedling roots gradually decreased in the L1, L2, and L4 treatments, and the root soluble sugar, NSC, and soluble sugar/starch ratios were higher in the L3 treatment than in the other treatments (**Figure 8**). The NSC (and its fractions) of the roots of 3-year-old *P. yunnanensis* seedlings did not significantly differ in the L1 treatment and the CK, and it was higher in the L1 treatment than in the L2, L3, and L4 treatments. The root NSC (and its components) of 3-year-old *P. yunnanensis* seedlings did not change significantly among the L2, L3, and L4 treatments, and the root soluble sugar/starch ratio did not change significantly among the L2, L3, and L4 treatments. This indicates that 1-year-old *P. yunnanensis* seedlings were more dependent on NSC in response to the shade treatment, indicating that the above-ground parts are required for the transport of photosynthetically assimilated substances to the root system, and that the root vigor was greatest in the L3 treatment, which was manifested by the maximum soluble sugar/starch ratio. This also suggests that 3-year-old *P. yunnanensis* seedlings have superior regulatory mechanisms to reduce their dependence on short-term carbohydrates in the shade treatment.

The root C content of 1-year-old seedlings significantly decreased under shade treatment, and the C content of 3-year-old seedlings increased in the L4 treatment; this may be related to the strategy employed by 3-year-old seedlings, wherein root C structure is optimized to maintain survival under shade treatment, which reflects their shift from a rapid growth strategy to a survival strategy (Kobe et al., 2010). The elevated root N content of 1-year-old seedlings under shade (L1, L2, and L4) possibly compensated for the light energy deficit by enhancing N uptake to support chlorophyll synthesis. In contrast, the root N content was lowest in the L3 treatment; the C/N ratio was significantly higher in the L3 treatment than in the CK, and the N/P ratio was lowest in the L3 treatment, suggesting that resources tended to be allocated to organic matter produced by C metabolism to sustain plant growth and development and to maintain the requirements for normal physiological functioning (Monson et al., 2021).

The N content of 3-year-old seedlings was generally lower in the shade treatments (L1, L3, and L4), which reflects adaptive adjustments to low light or N transport to aboveground parts; this suggests that 3-year-old seedlings rely more on C reserves than N metabolism under low light. Consistent with the ‘C storage preference hypothesis’ proposed by He et al. (2024) that mature trees tend to allocate C to structural components (e.g., lignin) to enhance tissue resistance under stress rather than to temporary metabolic reservoirs (NSC), the root P content of the 3-year-old seedlings was significantly reduced under shade, whereas the root P content of the 1-year-old seedlings was only elevated under extreme shade (L4), suggesting that young seedlings may maintain energy metabolism by increasing P uptake under low light conditions (Liu et al., 2024).

### 4.3 Adaptive strategies of *P. yunnanensis* seedlings to shade conditions

The phenotypic plasticity index values of the root morphological characteristics (e.g., root biomass, root surface area) and root C/N ratio indices were large in the 1-year-old seedlings. The biomass of each organ was significantly and positively correlated with the root starch, root NSC, and root C content, as well as the root C/P ratio (**Figure 11A**), indicating that the growth of young seedlings depended on the storage and transport strategy of photosynthetic assimilates in the roots; the root biomass ratios were not significantly different in the L1, L2, and L3 treatments, and the root index values and C content were reduced in these treatments. This indicates that changes in the biomass of 1-year-old *P. yunnanensis* seedlings under shade conditions were consistent with the NSC storage strategy of the roots, changes in the C content, and the root C/P ratio. Seedlings enhanced resource capture by maintaining the root biomass ratio and root mean diameter, and the conversion of starch to soluble sugar was stable, which supported aboveground growth.

The phenotypic plasticity index values for root stoichiometric characteristics (N/P, C/N, and N), soluble sugar/starch ratio, and soluble sugar content of 3-year-old seedlings were high. The biomass of each organ was significantly and positively correlated with root soluble sugar, soluble sugar/starch, root N content, and N/P ratio and negatively correlated with the P content (**Figure 11B**), suggesting that 3-year-old seedlings optimize the efficiency of resource use via alterations of C-N metabolism (decreasing the C/N ratio), maintaining starch storage (the root-shoot ratio was maintained in the L1, L2, and L3 treatments and increased in the L4 treatment), and increasing aboveground biomass investment (increasing the needle biomass ratio and maintaining the stem biomass ratio) in response to shaded environment. Three-year-old seedlings depend on N uptake and storage, and the absorption of N by the roots enhances the plant’s ability to withstand shade, suggesting that N fertilizer could be applied to 3-year-old seedlings under shaded conditions.

## 5 Conclusion

Our findings indicate that *P. yunnanensis* seedlings use age-related strategies to cope with shade stress, which reflects a trade-off between growth and survival. First, 1-year-old seedlings prioritized rapid biomass accumulation by increasing N uptake and maintaining root starch reserves, but they were at risk of C depletion under extremely shaded conditions. Their high plasticity in root morphology (e.g., surface area) and C/P regulation supports their transient growth but limits their long-term shade tolerance. Second, 3-year-old seedlings adopt conservative strategies to reallocate C to structural components and optimize C:N:P ratios to enhance stress tolerance. Their dependence on soluble sugars rather than starch and their suppression of N metabolism suggest that metabolic efficiency is prioritized over growth. Third, phenotypic plasticity diverges sharply with age: 1-year-old seedlings rely on morphological adjustments, whereas 3-year-old seedlings utilize stoichiometric flexibility. Our results suggest the need for age-specific management: moderate shade (30–80% light) favors root development in 1-year-old seedlings, and 3-year-old seedlings can tolerate greater shade (5–45% light) under N supplementation. Long-term field experiments and isotope tracing are needed to validate C-nutrient redistribution pathways to enhance our understanding of ontogenetic ecological niche differentiation during forest regeneration.

## Data Availability

The original contributions presented in the study are included in the article/supplementary material. Further inquiries can be directed to the corresponding author.
